# Single Person Identification and Activity Estimation in a Room from Waist-Level Contours Captured by 2D Light Detection and Ranging

**DOI:** 10.3390/s24041272

**Published:** 2024-02-17

**Authors:** Mizuki Enoki, Kai Watanabe, Hiroshi Noguchi

**Affiliations:** 1 Graduate School of Engineering, Osaka City University, Osaka 558-8585, Japan; 2 Graduate School of Engineering, Osaka Metropolitan University, Osaka 558-8585, Japan

**Keywords:** LIDAR, deep learning, action recognition

## Abstract

To develop socially assistive robots for monitoring older adults at home, a sensor is required to identify residents and capture activities within the room without violating privacy. We focused on 2D Light Detection and Ranging (2D-LIDAR) capable of robustly measuring human contours in a room. While horizontal 2D contour data can provide human location, identifying humans and activities from these contours is challenging. To address this issue, we developed novel methods using deep learning techniques. This paper proposes methods for person identification and activity estimation in a room using contour point clouds captured by a single 2D-LIDAR at hip height. In this approach, human contours were extracted from 2D-LIDAR data using density-based spatial clustering of applications with noise. Subsequently, the person and activity within a 10-s interval were estimated employing deep learning techniques. Two deep learning models, namely Long Short-Term Memory (LSTM) and image classification (VGG16), were compared. In the experiment, a total of 120 min of walking data and 100 min of additional activities (door opening, sitting, and standing) were collected from four participants. The LSTM-based and VGG16-based methods achieved accuracies of 65.3% and 89.7%, respectively, for person identification among the four individuals. Furthermore, these methods demonstrated accuracies of 94.2% and 97.9%, respectively, for the estimation of the four activities. Despite the 2D-LIDAR point clouds at hip height containing small features related to gait, the results indicate that the VGG16-based method has the capability to identify individuals and accurately estimate their activities.

## 1. Introduction

Socially assistive robots are anticipated to find application in monitoring the health and accidents of older adults and providing psychological support in their homes [1]. Typically, these robots have the ability to engage in conversations with older adults at appropriate times, accompanied by various motions. For effective communication, it is desirable for socially assistive robots to detect residents’ locations and daily actions accurately and then engage with older adults with greetings.

Cameras are frequently used as sensors for location and action detection owing to their easy installation in commercially available robots. However, the use of cameras may invade privacy, particularly in home environments where residents engage in personal activities. Additionally, the horizontal view angle of the camera installed in the robot is usually limited, which is insufficient to cover the entire room. On the other hand, mobile robots often incorporate 2D Light Detection and Ranging (2D-LIDAR) for localization and environment recognition. The 2D-LIDAR measures object horizontal contours by rotating the distance-measuring laser, allowing them to capture distance information with a horizontal view of more than 180 degrees. This broad view easily covers the entire room with just one LIDAR. Importantly, 2D-LIDAR only captures human contours without violating privacy issues. This LIDAR can be easily integrated into socially assistive robots because they have become smaller and less expensive in recent years. Therefore, LIDAR may be a suitable choice for socially assistive robots to detect residents.

Numerous studies have explored the measurement of human locations using LIDAR. Person tracking has been achieved through various approaches, such as employing multiple fixed 2D-LIDARs positioned at ankle height [2], as well as utilizing a single 2D-LIDAR mounted on a mobile robot [3]. Multiple fixed 2D-LIDARs at hip height were used to track multiple people in a building lobby [4], and resident trajectories were captured [5]. In recent years, 3D-LIDAR has become prevalent, capable of capturing 3D point clouds by measuring multiple layers of 2D data. Consequently, 3D-LIDAR is also employed for person tracking, as demonstrated in studies using either 2D- or 3D-LIDAR [6]. Papers such as [7,8] achieved person tracking using single 3D-LIDAR. To enhance multiple people tracking, research involving both 3D-LIDAR and RGBD cameras has been explored [9].

Traditionally, LIDAR was limited to person tracking, but recent research has successfully measured more information than just person locations. Notably, LIDAR can extract sufficient features from contour point clouds [10]. Person identification has been realized using both 2D-LIDAR [11] and 3D-LIDAR [12,13,14]. Combining 2D LIDAR and cameras has also proven effective for person identification [15]. LIDAR has shown potential beyond location measurement and person identification, with studies attempting other estimation tasks. Examples include age and height estimation using 2D-LIDAR [16] and age and sex estimation using 3D-LIDAR [17]. Additionally, action recognition using LIDAR has been explored, with notable examples such as fall detection using a single 2D LIDAR [18], behavior recognition from extracted trajectories using 2D LIDAR [19], and action recognition using the combination of 3D-LIDAR and a mm-Wave sensor [20]. Hence, LIDAR exhibits substantial potential for realizing location measurement, person identification, and action estimation of residents in a room.

Many research efforts focused on estimation using LIDAR often leverage 3D-LIDAR due to its ability to measure dense and numerous point clouds, capturing features related to a person. However, 3D-LIDAR is costly. When considering the application in socially assistive robots, 2D-LIDAR emerges as a suitable choice due to its affordability and low power consumption. Fortunately, the previous studies mentioned above have indicated that 2D-LIDAR demonstrates sufficient performance to capture resident locations.

Although 2D-LIDAR captures less data for person identification and action estimation compared to 3D-LIDAR, research using 2D-LIDAR at ankle height has successfully achieved person identification. The sequence of ankle contours reflects gait features adequately [11,18]. Person identification research using 3D-LIDAR also suggests that the lower part of the body improves identification performance [13]. This implies that person identification could be realized to some extent using 2D-LIDAR if gait features are captured.

However, 2D-LIDAR measurement at ankle height in a home environment is not suitable due to obstacles such as desk legs and luggage, causing potential occlusion. Considering integration into socially assistive robots, the robot’s height would be at tabletop height for communication with residents. Although person identification using 2D-LIDAR at hip height is challenging, the hip contour can provide supportive features indirectly representing individual characteristics such as arm swing, torso rotation, and gait features. Fortunately, since the people to be identified are limited to a few individuals, such as family members and caregivers, identifying three or four people using only hip contours may suffice.

Regarding action estimation, previous research has successfully estimated actions from 2D-LIDAR vertical contours [21], suggesting the potential to estimate a few actions from hip contours captured by 2D-LIDAR. Therefore, person identification and action estimation may be feasible using 2D-LIDAR at hip height.

The conventional method for estimating from 2D LIDAR data typically employs traditional machine learning techniques based on features extracted from point clouds of human contours [3,21]. In recent times, as neural networks have advanced, estimation methods increasingly utilize deep learning approaches. Given that gait and actions involve time-series changes, methods employing deep learning often incorporate specialized networks for time-series learning. A typical example is the use of Long Short-Term Memory (LSTM) networks, frequently applied for learning from sequential LIDAR-based point clouds [13,18,19].

Another strategy involves projecting the sequence of point clouds into an image and estimating the person and activity from the projected images using deep learning methods. This approach benefits from the effectiveness of Convolutional Neural Networks (CNNs) in object recognition from images. For instance, leg contours were detected from an occupancy map created from 2D-LIDAR point clouds [22]. In [11], an image was superimposed on a point cloud of a person walking, and person identification was performed using a CNN model for the superimposed images.

Since it remains unclear which approach is superior for person identification and action estimation from hip contour data captured by 2D-LIDAR, there is a need to evaluate the performance of both LSTM-based and CNN-based approaches.

The purpose of this study is to develop methods for person identification and action estimation from hip contours captured by 2D-LIDAR in a room. Two methods using LSTM and CNN models are compared. As previously mentioned, the goal is to identify a maximum of four people, considering measurements in a home environment for socially assistive robot use. Given the difficulty in estimating complex actions from hip contours, four simple actions are selected for activity recognition: walking, standing, sitting, and opening/closing a door.

## 2. Single Person Identification and Activity Estimation Method

**Figure 1:**
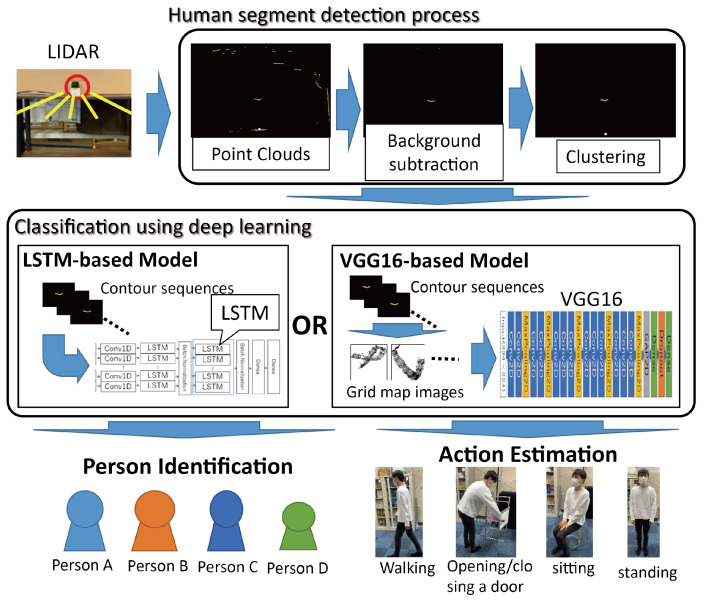
Method overview.

The overall structure of estimation methods is shown in Figure 1.

### 2.1. Used 2D LIDAR

The 2D-LIDAR employed in this study was the URG-04LX from Hokuyo Automatic Co., Ltd., Osaka, Japan. This choice was driven by its relative affordability, compact size, and ease of integration into socially assistive robots. The device operates at a scanning sampling rate of 10 Hz, offering a scanning angle of 240 degrees with an angular resolution of 0.36 degrees. The maximum detection distance of the device is 5.6 m. Given these specifications, a single device strategically placed on one side of the room was sufficient to cover the entire area, making it a suitable choice for the measurement purposes of this study.

### 2.2. Extraction of Person Contour

Before performing person identification and activity estimation, it is crucial to detect the human contour from the 2D-LIDAR scan point clouds. Various methods exist for extracting human contours from 2D-LIDAR data, including machine learning-based detection [22] and clustering techniques [23]. Fortunately, as the point cloud segment of the hip contour is extracted from the 2D-LIDAR at hip height, distinguishing these segments from others is more straightforward compared to ankle segments. Therefore, a simple approach was employed using background subtraction and clustering. Additionally, for human localization, tracking methods are sometimes employed to achieve smooth position estimation. However, in our scenario, achieving the smooth position is not crucial as accurate contour point clouds are the primary requirement for identification and estimation. Consequently, we solely relied on the contour extraction method without employing any filtering or tracking techniques.

**Figure 2:**
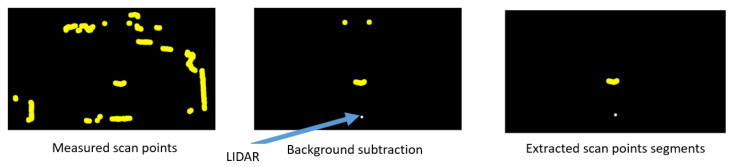
Preprocessing flow of 2D-LIDAR data.

Specifically, the detection of the hip contour was conducted as follows. The example point clouds are illustrated in Figure 2. Room contours were pre-measured as background data for 5 min, and the average distance for each scan angle was used as the background distance. The threshold for the foreground decision was set at 0.015 m, based on the results of a pretest. Points in front of the background distance with the threshold were considered foreground points. To identify human contour segments, foreground points were clustered using Density-Based Spatial Clustering of Applications with Noise [24], a technique often employed for 2D-LIDAR point cloud segmentation. Through preliminary tests, the parameter epsilon was set to 0.1 m, and the number of minimum configuration points was set to 10. This configuration ensured robust extraction of the human hip contour without missing segments.

To preliminarily assess the feasibility of position localization using a simple person contour detection method, data were collected while walking around the room for approximately 20 s with reference data using an optical motion capture system. Using this collected data, the efficacy of the person contour detection method was examined to determine if it could successfully detect a target person in each frame. It was confirmed that detection was achieved in all frames. Additionally, the root-mean-square error was calculated to be 0.286 m. This error was considered to arise from estimating the center of the contour rather than the center of the body. However, since both subsequent methods only require rough body position as input, it was considered that they have sufficient performance.

### 2.3. LSTM-Based Model

As mentioned in the introduction, LSTM networks have been commonly utilized for learning from sequential LIDAR-based point clouds due to their ability to handle time-related sequences. Consequently, we opted for LSTM as one of the models for identification and recognition. LSTM addresses the gradient loss problem encountered in Recurrent Neural Networks and is a widely used deep learning model in natural language processing and time-series data applications. With three gates (forget gate, input gate, and output gate), LSTM is known for its ability to handle past time-series data, making it advantageous for learning sequential patterns.

**Figure 3:**
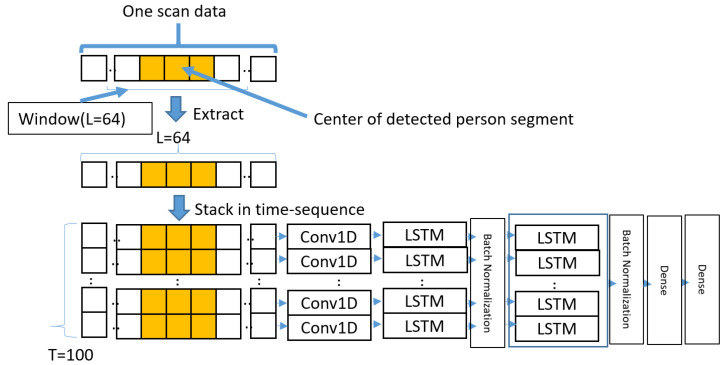
Network structure of LSTM-based model.

In our case, the LSTM network was constructed by referencing a deep learning model that utilized LSTM to identify a person from ankle contours [18]. The network structure is depicted in Figure 3. For each frame, a window of 64 data points around the center index of the detected person segment was extracted from the distance data (726 data points). Preliminary tests indicated that approximately 25 degrees of data points around the person contour were needed to cover the torso in the room. Therefore, the window size was defined as a power of 2 to achieve this coverage angle.

This process was repeated for 100 frames (equivalent to 10 s based on the scanning sampling rate of the LIDAR), and the resulting 100-frame data was stacked into input data with a size of 100 × 64. This number of frames for input was equivalent to the value in the previous research [18]. After convolution with a 1D convolutional layer (conv1D) using a kernel size of 8 for each frame, the data were input into an LSTM with two stages. Finally, the dense layer was convolved twice to produce the estimated result. For the deep learning parameters, the loss function employed was categorical cross-entropy, and the optimization function used was the Adam function with a learning rate of 0.0001.

### 2.4. VGG16-Based Model

The alternative method involved generating an image from a point cloud and utilizing a CNN-based image classification approach for the generated image. The image generation method followed a similar approach to gridmaps used in previous studies [11,22]. Specifically, scan points were projected onto a map with a 0.01 m grid on each side. Additionally, a binary image was constructed by overlapping 100 frames, akin to the LSTM model, representing time information. However, this process introduced a challenge as the generated image size varied based on factors such as the person, movement speed, and actions.

**Figure 4:**
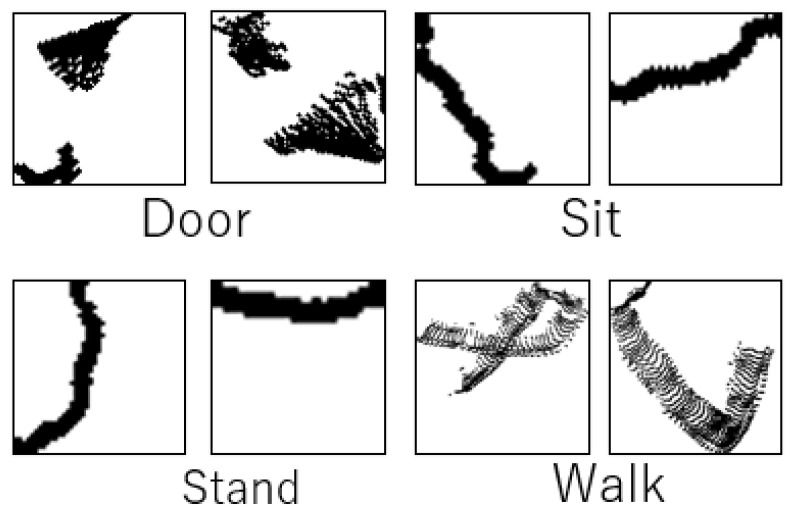
Typical gridmap images of each activity.

To address this issue, after generating the image, it was cropped using a bounding rectangle that included only filled grids. Subsequently, the cropped image was resized into a 224 × 224 pixel image based on the longest side of the bounding box rectangle area, ensuring uniformity across all images. Typical images for action estimation are illustrated in Figure 4. This image served as the input for the deep learning model.

**Figure 5:**
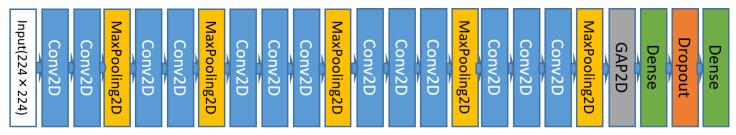
VGG16 structure.

For the estimation from the generated images, VGG16 [25] was employed. This method has traditionally been used as a CNN-based image classification approach. VGG16, while an early CNN-based image classification model, is known for its stable and relatively good image-recognition performance. Therefore, it was selected as a suitable choice for the initial trial. VGG16, as its name implies, comprises 16 layers, as illustrated in Figure 5. Its structure is characterized by multiple convolutions with 3 × 3 filters. Due to the limited size of the dataset for image classification, pretrained weights from ImageNet were used for model learning through transfer learning. In the model, the first dense layer in the fully connected layers uses the ReLU activation function, while the second dense layer for the final output employs the softmax function.

Since each captured contour strongly depends on the orientation from the person to the LIDAR, potentially affecting performance, images were randomly rotated by −45 to 45 degrees during the training phase as an augmentation technique to avoid overfitting. The loss function and optimization function were set the same as in the LSTM-based model.

## 3. Experiment on Person Identification and Action Estimation

### 3.1. Dataset

**Figure 6:**
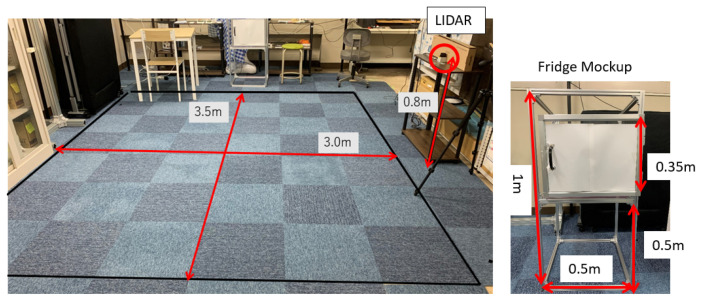
Experiment room setting.

The dataset measurements were conducted in a space simulating a resident room, as depicted in Figure 6. Four healthy male individuals participated in the experiment. The characteristics of each participant are detailed in Table 1. Participants were instructed to walk freely without specific guidelines regarding starting position, direction, walking route, or speed.

**Figure 7:**
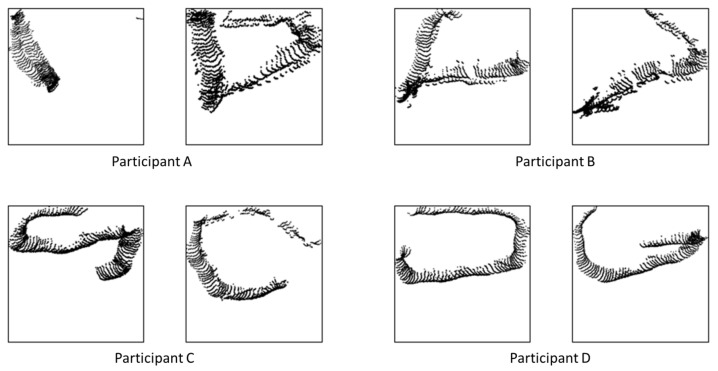
Typical walking gridmap images of each participant. Participant A through D correspond to the participants listed in Table 1.

Walking data were measured for each of the four participants, with a duration of 1800 s per participant. Typical images for the VGG16-based model, created from the measurement data, are presented in Figure 7.

For action estimation, sitting, standing, and door opening/closing actions were measured individually. Sitting actions involved participants sitting in a chair without moving for the entire 10 s duration. Participants were instructed to randomly change the location and orientation of the chair in each trial. The total measurement time for sitting actions was 6000 s across all participants. Similarly, standing postures were measured for 10 s without any movement. The total measurement time for standing actions was 6000 s across all participants. For door opening/closing action measurements, a mockup of a refrigerator was prepared, as shown on the right side of Figure 6. The opening/closing actions were performed during a 10 s period, similar to sitting and standing actions. The mockups were rotated and moved randomly in each trial. The total measurement time for door opening/closing actions was 6000 s.

### 3.2. Evaluation Method

For both the LSTM-based model and the VGG16-based model, the data were segmented into 10 s windows, aligning with the input requirements of both models. Thus, the total dataset for person identification comprised 170 windows × 4 persons, resulting in 720 windows. To mitigate data bias in the analysis, the data were randomly split into training and test sets in a 9:1 ratio while maintaining an equal proportion of the four individuals.

Similarly, for action estimation, the data were preprocessed by dividing them into 10 s windows, resulting in 2400 windows. Subsequently, the data were divided into training and test sets in a 9:1 ratio, maintaining an equal proportion for the four actions.

Regarding the parameters used in deep learning training, both LSTM and VGG16 were trained for 100 epochs. The batch size for LSTM was set to 32, while the batch size for VGG16 was set to 16, considering the deep learning model size and GPU memory constraints. It was confirmed that the model converges sufficiently within this number of epochs in both models.

Since the estimation performance was expected to depend on the number of people for identification, both three-person and four-person identifications were conducted. In the three-person identification, all four combinations of the three choices were estimated, and the calculated results were averaged. The evaluation method for person identification and action estimation was accuracy, calculated as the overall percentage of correct answers. This metric provides a straightforward representation of overall performance in a multiple-class estimation problem.

### 3.3. Experiment Results and Discussion

The results are presented in Table 2. The VGG16-based model exhibited superior performance compared to the LSTM-based model. Both models can handle time series properties and torso contour information. However, the LSTM-based model is more focused on features related to the time series due to its network structure, while the VGG16-based model is considered to be more attentive to features associated with torso contours, given the nature of the generated images. The outcomes suggest that the VGG16-based model may be better suited for person identification and action estimation based on torso contour information captured by 2D-LIDAR at hip height. Additionally, the VGG16-based model utilized transfer learning data, which could enhance the model’s ability to capture contour features and potentially contribute to achieving better performance.

The action estimation results demonstrated better performance than person identification. This heightened performance could be attributed to the fact that the four selected actions in this study were easily distinguishable from each other, considering the distinctiveness of their contours and their time-series information. The detailed classification of behaviors and similar actions remains a future consideration.

In a previous study using 2D-LIDAR at ankle height, 92.4% accuracy was achieved for three-person identification [18]. Another study realized 98% accuracy in 29 person identification using multiple 2D-LIDARs at ankle height [11]. In comparison, the accuracy of person identification in our methods was slightly lower. In discussions on gait recognition based on 3D-LIDAR measurements [13], it is suggested that the ankle contour is more advantageous for individual identification than the waist contour. This is because the waist contour may not exhibit significant individual variation in arm swing, and it sometimes includes non-periodic arm movements such as touching the face. Despite the lower performance in this study compared to previous ones, the method did not experience a dramatic decrease in accuracy. Even when the ankles are invisible, the visible contours of the waist and the swing of the arms represent gait-related features. The deep learning method successfully captured these subtle features, indicating the potential for individual identification.

As for action estimation, a study using 2D-LIDAR at ankle height achieved an estimation accuracy of 92.3% for four types of actions (walking, standing, sitting, and falling) [18]. The results in our study surpassed this previous study. For particular actions (opening/closing doors and sitting), information on arm contours could support distinguishment among actions, which might improve estimation accuracy.

Furthermore, apart from the issue of the performance, the VGG16-based model has the advantage on application. The 2D-LIDARs vary in characteristics such as distance resolution and angular resolution. In the case of an LSTM-based model, the input vectors would need to be adjusted according to these specifications of 2D-LIDAR, and this adjustment requires retraining. However, with the VGG16-based model, since the inputs are projected onto images, the same training data could potentially be used even if a different LIDAR is employed. Therefore, the VGG16-based model may reduce dependency on specific LIDAR characteristics, enabling person identification and action recognition.

## 4. Extra Experiment on VGG16-Based Model

Following the previous experiment, further validation was conducted on the VGG16-based model, because the VGG16-based model would be promising for person identification and action recognition.

### 4.1. Experiment on Comparison of CNN Methods

As a first step, comparative experiments were conducted regarding the structure of image recognition models. Generally, in image recognition, it is known that increasing the complexity of the network can improve performance. Therefore, experiments were conducted to investigate how much performance improves when using deep learning methods with network structures that are deeper and have a larger number of parameters than VGG16.

For the experiment, two models were employed: VGG19, which is three layers deeper, and ResNet50 [26], a deeper model developed later with a higher number of parameters. Both models are commonly used methods in deep learning for traditional image recognition. The datasets, parameters, and evaluation methods remained identical to those used with VGG16 in the previous experiments. However, for person identification, only four-person identification was tried, because four-person identification is more challenging task than three-person identification.

The results are presented in Table 3. As shown in the table, there was a slight improvement in performance with increasing model complexity, but there was no dramatic improvement. It is presumed that the performance of action recognition remained unchanged as recognition accuracy was already sufficiently high. For person identification, a slight improvement in performance was observed. However, interestingly, the simpler VGG19, which is only deeper than VGG16, performed better than ResNet50 which is a large model in terms of both complexity and parameter count. This suggests that simply increasing the number of parameters may not necessarily lead to improved performance. Various CNN-based image recognition methods have been developed, and exploring the application of these methods and searching for network models tailored to the current dataset are future tasks.

### 4.2. Experiment on Time Dependence

The performance of the VGG16-based model in three-person identification was slightly lower than that in four-person identification, which requires further experiments. In addition, considering real-world recognition scenarios, it is desirable to reduce the number of frames used for estimation. Therefore, additional experiments were conducted on the VGG16-based model, which achieved good performance, to examine the estimation performance according to the number of frames. In these experiments, the time interval in one window was varied from 2, 4, 6, and 8 to 10.

However, since the estimation performance could be dependent on the number of datasets, the number of training and test data in the experiment was limited to the same number as in the previous experiment (10 s experiment) by randomly sampling from the entire dataset.

**Figure 8:**
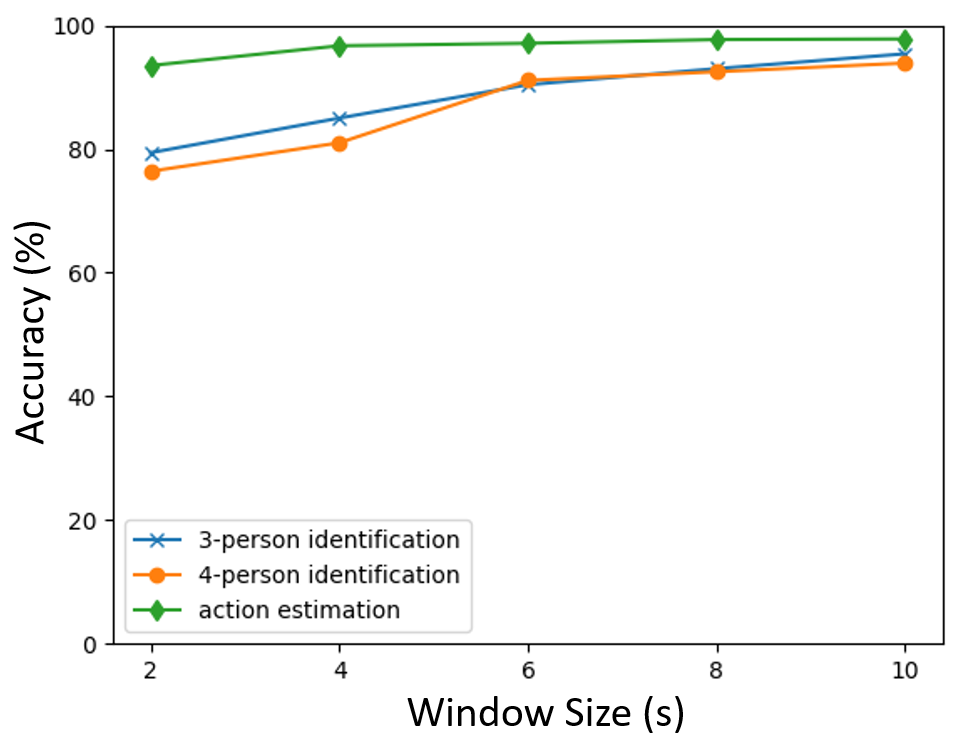
Estimation score with window size.

The results are illustrated in Figure 8. The findings indicated that the number of frames was related to performance, but the difference was small in action estimation. The dataset included static postures such as sitting and standing, where time-series information is not as crucial for estimation. This characteristic might reduce the dependency on the window size in action estimation. However, the duration of actions naturally varies depending on the specific action. Therefore, if additional actions are included, their durations would need to be adjusted accordingly. For instance, reducing the action such as opening/closing the door might enable accurate estimation even with shorter durations. Investigating the relationship between the duration required for estimation and the type of action, as well as exploring methods to handle them variably, remains a future research direction.

On the other hand, the performance of person identification gradually decreased as the number of frames was reduced. The figure illustrates that the performance starts to decrease significantly and drastically from a 6 s window size. The VGG16-based model utilized images created from 2D-LIDAR point clouds in walking sequences, which included not only torso contour changes but also movement-related information such as movement speed. Person identification would require time series-related features, such as gait. A smaller window size could lead to a shortage of time series-related features, resulting in performance decrease. In other words, since a certain length of window would be needed to capture these features, a 6 s window size might be required for person identification from 2D-LIDAR data at hip height. There was little difference between three-person and four-person identification, indicating that the deep learning model had the capability to obtain sufficient features to identify a small number of people.

## 5. Conclusions

In this study, we proposed methods for person identification and action estimation using 2D-LIDAR data placed at hip height. The methods rely on LSTM and VGG16, which are deep learning techniques. In the experiment, three and four people were identified, and four actions (sitting, standing, walking, and opening/closing a door) were estimated. The VGG16-based model achieved approximately 90% accuracy in person identification and 97% accuracy in action estimation. The 6 s window interval maintained sufficient performance for both person identification and action estimation. The results suggest that a single 2D-LIDAR at hip height might possess adequate performance to be utilized as a sensor for identifying residents and estimating actions in a room.

## Figures and Tables

**Table 1:** Characteristics of participants.

	Height (cm)	Weight (kg)	Waist Circumference (cm)
Participant A	175	55	71
Participant B	180	70	75
Participant C	175	76	85
Participant D	170	70	78

**Table 2:** Result of person identification and action estimation.

Model	3-Person Identification	4-Person Identification	Action Estimation
LSTM-based model (%)	75.9	65.3	94.2
VGG16-based model (%)	88.1	89.7	97.9

**Table 3:** Result of comparison among recognition methods.

Model	4-Person Identification	Action Estimation
VGG16-based Model (%)	89.7	97.9
VGG19-based Model (%)	94.2	97.0
ResNet50-based Model (%)	90.8	98.1

## Data Availability

There are no open data related to this research because informed consent did not include data opening.
